# Chromatographic fingerprints analysis and determination of seven components in Danmu preparations by HPLC–DAD/QTOF-MS

**DOI:** 10.1186/s13020-020-00301-5

**Published:** 2020-02-18

**Authors:** Nanxin Li, Jinju Zhang, Ying Zhang, Zhiguo Ma, Jinhong Liao, Hui Cao, Menghua Wu

**Affiliations:** 1 College of Pharmacy, Lingnan Traditional Chinese Medicine Research Center of Jinan University, Lingnan Branch of National Engineering Research Center for Modernization of Traditional Chinese Medicine, Jinan University, Guangzhou, 510632 Guangdong China; 2 Guangdong Provincial Key Laboratory of Research on Pharmacodynamic Substances and Innovative Drugs of Traditional Chinese Medicine, Guangzhou, 510632 Guangdong China; 3 Hainan Senqi Pharmaceutical Co., Ltd, Haikou, 570216 Hainan China

**Keywords:** Chromatographic fingerprint, Quantitative analysis, HPLC–DAD/QTOF-MS, Danmu preparations

## Abstract

**Background:**

Danmu preparations (Danmu Capsule and Danmu Syrup), which are made from *Nauclea officinalis* stem extracts, have good clinical efficacy in acute tonsillitis, acute pharyngitis and upper respiratory tract infection. However, there is currently no reliable and systematic method to control the quality of these two Danmu preparations.

**Methods:**

Using high-performance liquid chromatography (HPLC) coupled with diode array detection (DAD), the fingerprints of the Danmu preparations were established at 250 nm to comprehensively investigate the stability of preparation process. The chemical constituents in the Danmu preparations were separated and identified by HPLC coupled with quadrupole-time-of-flight high-definition mass spectrometry (HPLC–Q-TOF-MS). And seven major components were simultaneously determined at dual wavelengths (250 nm, 326 nm).

**Results:**

The results of HPLC fingerprint similarity evaluation showed that the similarity values of 25 batches of Danmu preparations were more than 0.993. Twenty-three compounds, including 10 alkaloids, 6 phenolic acids, 2 iridoids, and 5 unknown compounds, were identified or tentatively characterized according to the retention times and MS/MS fragment patterns of compounds. The developed assay method of seven components was validated with acceptable linearity, precision, repeatability, stability and recovery. The contents of strictosamide belonging to alkaloids as the most abundant constituent in Danmu Capsule and Danmu Syrup were 43,681.20–99,652.49 μg/g and 1567.83–2427.25 μg/mL respectively. The contents of protocatechuic acid which were the highest in measured phenolic acids were 2633.01–7739.78 μg/g in Danmu Capsule and 192.05–448.71 μg/mL in Danmu Syrup, respectively. As an iridoid, the contents of sweroside in Danmu Capsule and Danmu Syrup were 1573.82–2789.81 μg/g and 70.32–182.81 μg/mL, respectively.

**Conclusion:**

The established qualitative analysis method of fingerprint can be used to attain standardization, uniformity and stability of the preparation process. Meanwhile, the quantitative analysis in this study can be used as an accurate assay method for preparations.

## Background

Danmu Capsule and Danmu Syrup, made from the development of the single medicinal materials of *Nauclea officinalis* (Pierre ex Pitard) Merr. et Chun, have been used clinically in the treatment of acute tonsillitis, acute pharyngitis, acute knot membrane inflammation and upper respiratory tract infection for many years [1, 2]. Across the world, the genus *Nauclea* is distributed in tropical Asia, Africa and Oceania. *Nauclea officinalis*, commonly known as Danmu, mainly grows in the forests of the medium altitude areas in southern China and has often been used as folk medicine by the Li Nationality [3, 4].

A number of components have been isolated and identified from *Nauclea officinalis*, including alkaloids, phenolic acids, iridoids, pentacyclic triterpenoids and flavonoids [5, 6]. In addition, some of the components showed significant biological activities. Strictosamide, the representative constituent of *Nauclea officinalis* and a kind of indole alkaloid, is confirmed to have antibacterial, antiviral, anti-inflammatory and analgesic activities [7–10]. Phenolic acids such as protocatechuic acid and chlorogenic acid have good antioxidation, anti-inflammatory and anti-microbial activities [11–13], while sweroside, an iridoid, provides liver protection and has analgesic and anti-inflammatory activities [14, 15], and these compounds are likely to be important pharmacodynamic components in the efficacy of Danmu and its preparations.

Thus far, there have been few studies on the quality control of Danmu Capsule and Danmu Syrup, and the quantitative analysis of these products is mainly based on the content of only strictosamide [4]. Under the national drug standard for Danmu preparations, only the ultraviolet spectrophotometric method is used to determine the total flavonoid content, which lacks accuracy. None of the above methods can reflect the true quality of Danmu Capsule and Danmu Syrup. Currently, a few analytical methods, including HPLC [16], UPLC [17] and LC–MS [18], have focused on the quality assessment of Danmu preparations (Danmu Injection and Danmu Tablet). However, the samples of Danmu preparations in these studies are relatively few, and there is no systematic and reliable method to control the quality of the other two Danmu preparations, capsules and syrup. In fact, there have been many reports about the safety of traditional Chinese medicine (TCM) injections in recent years [19, 20]. As oral preparations, capsules and syrup are more convenient and safer than injection. And Danmu Capsule and Danmu Syrup are more popular in clinical application. Therefore, it is imperative to establish an accurate and universal analytical method for the quality assessment of Danmu Capsule and Danmu Syrup.

The chromatographic fingerprints of traditional Chinese medicine represent a multi-indicator quality control method that can comprehensively reflect the chemical composition information in preparations. This method has become an increasingly popular Chinese medicine quality evaluation model at home and abroad [21, 22]. However, there are currently no reports on the establishment of chromatographic fingerprints for Danmu preparations. In addition, at present, the technology of HPLC–Q-TOF-MS is widely used in the analysis and identification of multiple components in TCM and compounds [22–24]. Through HPLC separation, the advantages of precise molecular weight determination by tandem high-resolution time-of-flight mass spectrometry are used to accurately identify multiple components of TCM. The combination of chromatographic fingerprints with qualitative and quantitative analysis can authentically, systematically and intuitively investigate the quality of Danmu preparations. Therefore, a reliable and practical method was established to realize both qualitative and quantitative analysis of Danmu Capsule and Danmu Syrup by HPLC–DAD/QTOF-MS in this study. The fingerprints of two Danmu preparations were established for the first time, and a total of seven main compounds were identified and accurately quantified. This method is useful and efficient and can be used as a reference for improving the quality standards of these two Danmu preparations.

## Methods

### Chemicals and materials

**Fig. 1:**
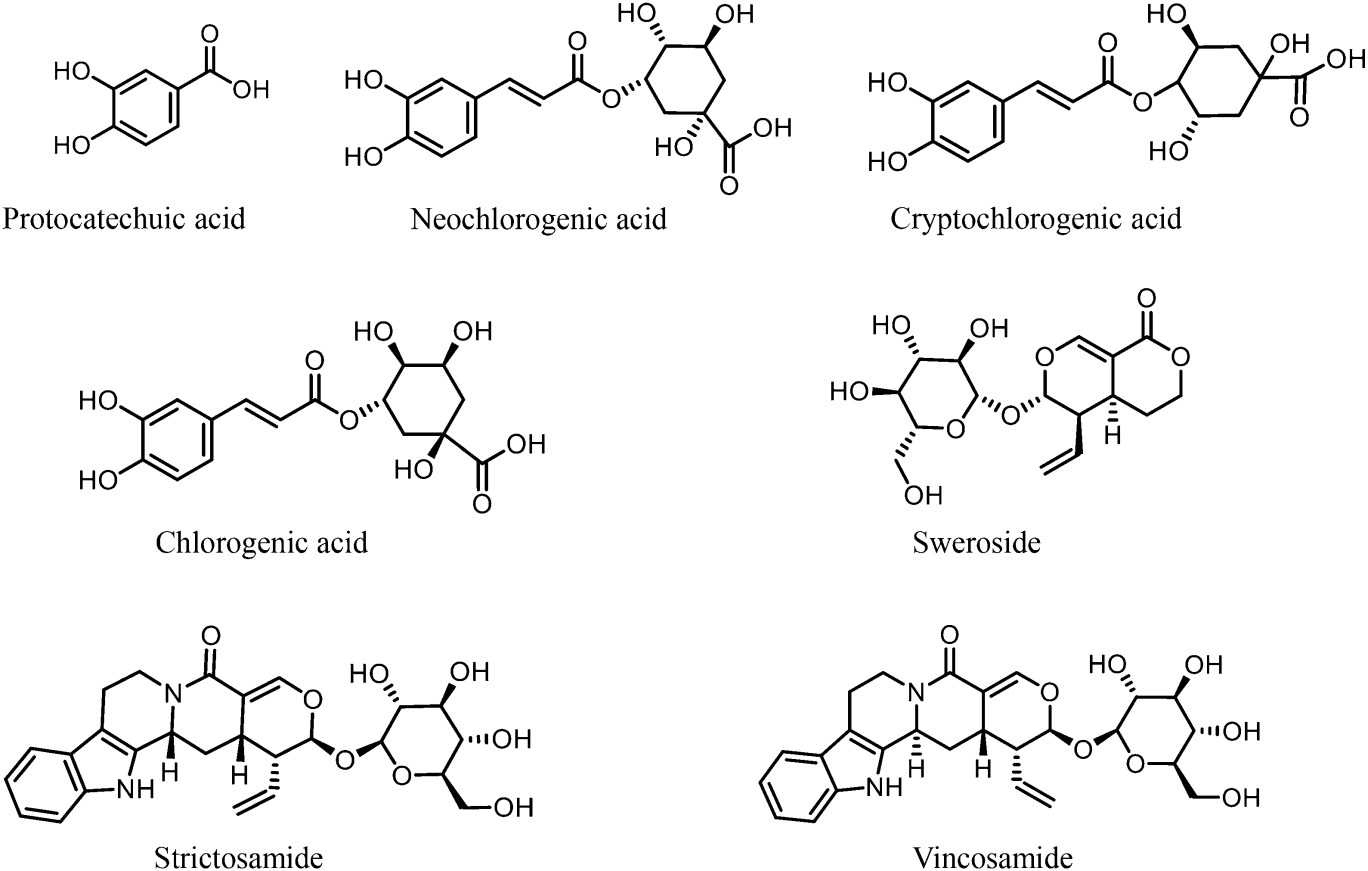
Chemical structures of the seven investigated compounds in Danmu preparations

The reference standards of protocatechuic acid (5809), neochlorogenic acid (6630), cryptochlorogenic acid (3208), chlorogenic acid (5077) and sweroside (3533) were purchased from Standard Technology Co., Ltd. (Shanghai, China). The strictosamide (111778-201102) was purchased from the National Institutes for Food and Drug Control (Beijing, China). The vincosamide (PRF10041624) was purchased from Biopurify Phytochemicals Ltd. (Chengdu, China). Their structures are shown in Fig. 1. The purity of all the reference compounds was determined to be ≥ 98% by normalization of the peak areas detected by HPLC–DAD, except for the content of strictosamide, which was calculated to be 95.3%.

The acetonitrile and formic acid were of HPLC grade and were purchased from Fisher Scientific (Fair Lawn, NJ, USA). All the other reagent solutions of analytical grade were supplied from General Reagent (Shanghai, China). The deionized water whose electrical resistivity was 18.2 μS/cm at 25 °C was obtained using a Milli-Q water purification system (Millipore, Billerica, MA, USA).

Twenty-five batches of commercially available Danmu Capsule and Danmu Syrup were collected from Hainan Senqi Pharmaceutical Co., Ltd. (Hainan China). The batch numbers are shown in Table 1.

**Table 1:** Contents of seven components in Danmu preparations (n = 2)

Sample	Batch number	Protocatechuic acid	Neochlorogenic acid	Cryptochlorogenic acid	Chlorogenic acid	Sweroside	Strictosamide	Vincosamide
Danmu Capsule (μg/g)	21181202	6256.70	2144.19	2493.95	2912.22	2789.81	99,652.49	2381.25
	21181203	5005.20	1776.46	2051.94	2388.15	2273.95	94,190.38	3571.03
	21190101	5138.88	2528.62	3173.23	3515.96	1869.90	49,347.16	2528.15
	21190102	6827.33	2923.81	3357.82	3977.83	1769.57	48,367.24	2534.48
	21190103	6243.84	2654.36	3033.11	3570.74	2058.60	48,666.45	2056.86
	21190301	2633.01	2279.71	2673.93	2857.39	1573.82	43,681.20	1303.58
	21190302	4785.44	1852.77	2253.44	2659.34	1767.13	65,450.28	3193.27
	21190303	3797.26	2817.05	3392.84	3828.06	1779.39	47,070.10	1777.36
	21190304	6327.20	2478.05	2921.73	3532.54	2176.98	56,793.52	2083.94
	21190305	3029.28	3021.76	3660.07	4076.78	1771.72	47,254.07	1780.27
	21190306	3688.83	2769.24	3404.15	3895.50	1786.12	48,215.87	1794.94
	21190307	7739.78	3301.36	4160.39	4667.69	1665.12	47,137.94	1568.89
Danmu Syrup (μg/mL)	11180806	289.92	76.84	69.59	80.99	109.50	1804.48	41.31
	11181102	348.23	106.90	96.31	116.16	102.65	1795.60	51.97
	11181105	265.32	142.30	136.40	177.67	107.94	2044.30	64.22
	11181109	448.71	66.28	59.96	68.20	70.32	2268.91	58.71
	11181117	326.55	117.92	106.22	137.15	182.81	2427.25	62.60
	11181201	304.12	108.91	96.94	118.19	173.47	2205.23	52.46
	11181210	364.39	94.82	93.46	120.30	88.88	1942.53	63.50
	11190101	239.90	84.34	84.29	105.59	133.98	1992.26	52.99
	11190308	233.50	116.30	128.74	146.95	77.99	1567.83	37.36
	11190412	192.05	105.98	112.98	130.47	94.14	1842.60	51.24
	11190502	348.84	149.27	150.62	192.39	114.49	2246.47	67.45
	11190504	276.74	145.62	144.03	200.24	154.04	2167.74	71.60
	11190506	260.44	167.89	166.43	222.83	114.64	2125.68	49.37

### Preparation of standard solutions

A mixed standard stock solution was prepared in methanol by accurately weighing seven reference substances. The stock solution was diluted with 50% methanol to obtain a series of working solutions with appropriate concentrations: 0.62–36.90 μg/mL for protocatechuic acid (1), 0.65–39.00 μg/mL for neochlorogenic acid (2), 0.70–42.00 μg/mL for cryptochlorogenic acid (3), 0.54–32.40 μg/mL for chlorogenic acid (4), 0.59–35.10 μg/mL for sweroside (5), 2.45–147.00 μg/mL for strictosamide (6) and 0.64–38.10 μg/mL for vincosamide (7). All the standard solutions were stored at 4 °C in the dark.

### Preparation of sample solutions

The powder of Danmu Capsule (0.1 g) was accurately weighted and extracted with 100 mL 70% methanol in an ultrasonic bath operating at 40 kHz with an output power of 300 W for 30 min. The sample solution was cooled to room temperature and adjusted to the original weight by adding extraction solvent. Each sample solution was filtered through a 0.45 μm membrane before being injected into the HPLC instrument for analysis.

A 1 mL solution of Danmu Syrup was accurately measured and was diluted to 25 mL in a volumetric flask with 70% methanol. Each sample solution was filtered through a 0.45 μm membrane before being injected into the HPLC instrument for analysis.

### Apparatus and chromatographic conditions

The chromatographic fingerprint and quantitative analyses were performed on an Agilent 1260 HPLC system (Agilent Technologies, Santa Clara, CA, USA) equipped with a vacuum degasser, a quaternary pump (G7111A), an autosampler (G7129A), a thermostatic column compartment (G7116A), and a diode array detector (G7115A, DAD). The separation was performed on a Waters X Select HSS C_18_ (5 μm, 4.6 × 250 mm) column. The column temperature was maintained at 35 °C. The mobile phase consisted of 0.1% formic acid aqueous solution (A) and acetonitrile (B) with a gradient program as follows: 0–2 min, 2% B; 2–20 min, 2–15% B; 20–35 min, 15–30% B; and 35–50 min, 30–38% B. The flow rate was kept at 1.0 mL/min. The injection volume was 10 μL. The detection wavelengths were 250 nm and 326 nm. The data were recorded and processed with OpenLab CDS 2 software.

The HPLC–Q-TOF-MS analysis had the same chromatographic conditions as the HPLC–DAD fingerprinting. The samples were separated in a Shimadzu Nexera Prominence LC system (Shimadzu, Kyoto, Japan) and analyzed in both positive and negative ion modes in an AB X500R QTOF (AB SCIEX, Framingham, USA). The ion source was electrospray ionization (ESI). The MS parameters in the negative ion modes were as follows: ion spray voltage, − 4500 V; ion source temperature, 200 °C; curtain gas pressure (N_2_), 30 psi; ion source gas 1, 50 psi; ion source gas 2, 60 psi; declustering potential, − 50 V; and collision energy, − 10 V. The parameters of the MS/MS scan mode were almost the same except that the collision energy was − 20 V and the collision energy spread was 5 V. The MS parameters in the positive ion modes were as follows: ion spray voltage, 5500 V; ion source temperature, 200 °C; curtain gas pressure (N_2_), 30 psi; ion source gas 1, 50 psi; ion source gas 2, 60 psi; declustering potential, 80 V; and collision energy, 10 V. The parameters of the MS/MS scan mode were almost the same except that the collision energy was 20 V and the collision energy spread was 8 V. The centroid mass spectra were acquired in the mass range of m/z 50–1000. The automatic acquisition mode was used to automatically select the strongest ion peak produced by the mass spectrometer as the parent ion of the second-order mass spectrum for fragmentation. The data were generated and analyzed on the SCIEX OS 1.3.1 software.

### Validation of the HPLC method

#### Linearity and sensitivity

A series of working solutions were injected into the HPLC. The calibration curves of seven targeted compounds were fitted based on the peak area (*y*) versus the concentration (*x*). The limit of detection (LOD) and limit of quantitation (LOQ) were estimated by signal-to-noise ratios of 3 and 10, respectively.

#### Precision, repeatability, stability and recovery

The intra-day and inter-day variations, which were used to evaluate the precision of the method, were determined by analyzing the standard solution at low, middle and high concentration levels within linear ranges five times on a single day and twice a day on 3 consecutive days. For the repeatability of the method, the six solutions prepared from the same sample were analyzed. For the stability of the method, the same sample solution was analyzed at 0, 2, 4, 8, 12 and 24 h. The variation in the peak area was used as the index for evaluating the precision, repeatability and stability and was expressed as the relative standard deviations (RSD). Recovery experiments were used to evaluate the accuracy of this developed method. Certain amounts of the mixed standard solution were spiked into the known amounts of sample in sextuplicate, and then the samples were extracted and analyzed by the proposed method. The recoveries were calculated according to the following formula: recovery (%) = (amount found − original amount)/amount spiked × 100%.

## Results

### HPLC fingerprint analysis

**Fig. 2:**
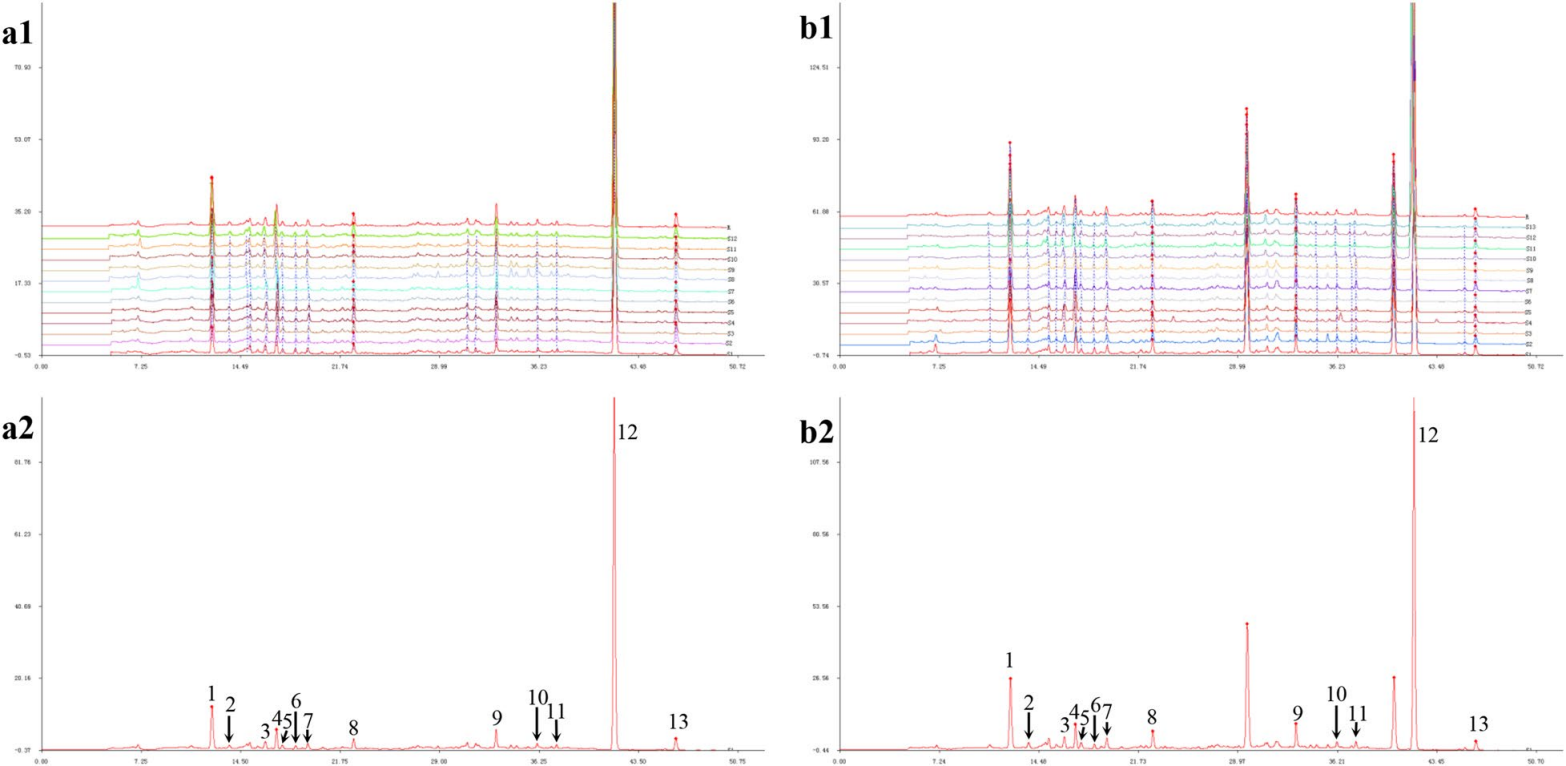
Overlapped chromatograms (**a1**, **b1**) and standard fingerprint (**a2**, **b2**) of Danmu preparations at a wavelength of 250 nm. **a** 12 batches of Danmu Capsule; **b** 13 batches of Danmu Syrup; (12) strictosamide

The similarity analysis was performed using professional software recommended by the State Food and Drug Administration: the Similarity Evaluation System for Chromatographic Fingerprint of TCM (version 2004A, State Pharmacopoeia Commission). The HPLC fingerprints of the different samples were standardized and established by selecting the common peaks in the chromatograms and normalizing the retention times of all the common peaks (Fig. 2). Peaks found in all samples with good stability and resolution were identified as common peaks and there were 13 common peaks in the HPLC fingerprints of Danmu Capsule and Danmu Syrup, respectively. The strictosamide (peak 12) was selected as the reference peak. The similarities between the chromatograms of the Danmu preparations was compared with their simulative median chromatograms generated as references. According to the cosine similarity given by the existing algorithm program, the similarity degrees of 12 Danmu Capsule were 0.999, 0.999, 1.000, 0.999, 0.999, 0.999, 0.993, 0.998, 0.998, 0.999, 0.996, and 0.998. The similarity degrees of 13 Danmu Syrup were 0.999, 0.997, 0.999, 0.993, 0.995, 0.998, 0.999, 0.999, 0.998, 0.997, 0.998, 0.998, 0.999. In summary, the similarity degrees of all samples were > 0.993, indicating that the intrinsic quality of the Danmu preparations was fairly stable.

**Fig. 3:**
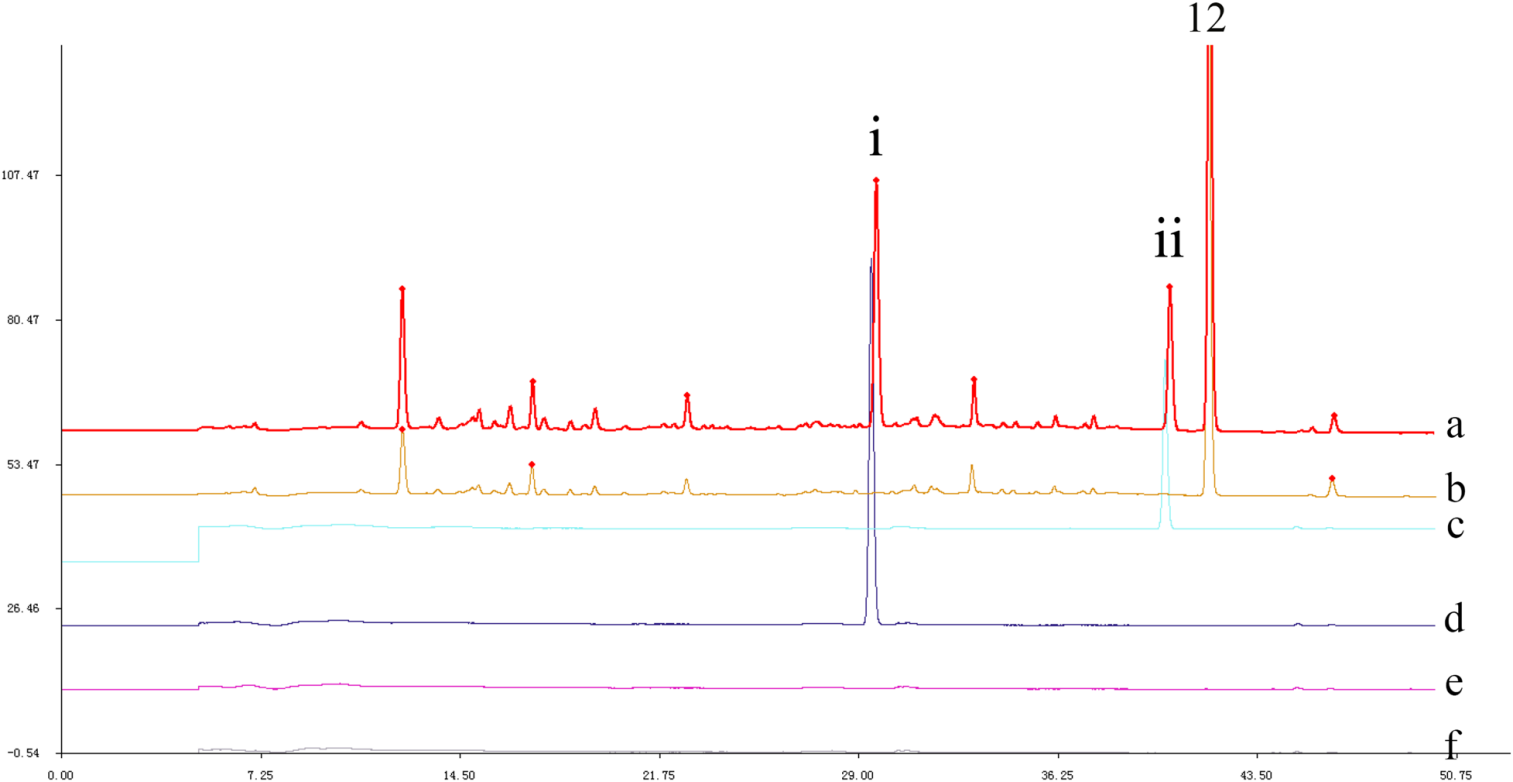
Comparation of HPLC chromatograms of Danmu preparations and its excipients. a–f Represented HPLC chromatograms of Danmu Syrup, Danmu Capsule, ethyl 4-hydroxybenzoate, sodium benzoate, sucrose and dextrin, respectively. (i) Sodium benzoate (ii) ethyl 4-hydroxybenzoate, (12) strictosamide

Compared with the typical fingerprint chromatograms for the capsules, there were two additional prominent peaks (i, ii) in the chromatogram of the syrup (Fig. 3). Therefore, we investigated the effects of the excipients of the two preparations on the peaks in the chromatogram. The main excipients of the syrup were sucrose, ethyl 4-hydroxybenzoate and sodium benzoate, and the excipient of the capsule was mainly dextrin. The negative reference solution was configured according to the preparation method of the test sample. The results suggested that peaks i and ii were sodium benzoate and ethyl 4-hydroxybenzoate, respectively, and that the sucrose and dextrin had no effects on the chromatographic peaks.

### Identification of constituents in the samples by HPLC–Q-TOF-MS

The optimized chromatographic and mass spectrometric conditions were used to analyze the mixed reference solution and the sample solution. Combined with the accurate relative molecular mass detected by the positive and negative ion mode, the exact molecular formula was calculated by the SCIEX OS 1.3.1 software within the range of 5 ppm mass deviation, and the compound was tentatively characterized by comparison with the molecular formula of the database compound. Then, a total of 23 compounds, including 10 alkaloids, 6 phenolic acids, 2 iridoids, and 5 unknown compounds, were identified or tentatively assigned according to the retention times and MS/MS fragment patterns of compounds. The results of identification were shown in Table 2.

**Table 2:** Identification of chemical constituents of Danmu preparations by HPLC-Q-TOF-MS

No.	t (min)	Molecular formula	Identification	[M+H]^+^/[M−H]^−^ (m/z)		Fragmentations (m/z)
				Measured mass (m/z)	Error (ppm)	
1^a^	3.954	C_7_H_12_O_6_	Quinic acid	191.0558 [M−H]^−^	− 1.6	127.0389, 109.0293, 93.0344
2	6.225	C_6_H_8_O_7_	Unknown	191.0195 [M−H]^−^	− 1.2	173.0085, 111.0089, 87.0090
3^a,d^ (peak 1)	17.046	C_7_H_6_O_4_	Protocatechuic acid	153.0194 [M−H]^−^	0.4	109.0294 [M−H−CO_2_]^−^, 91.0188 [M−H−CO_2_−H_2_O]^−^, 81.0350
4^a,d^ (peak 14)	18.787	C_16_H_18_O_9_	Neochlorogenic acid	353.0876 [M−H]^−^	− 0.6	191.0559 [M−H−C_9_H_6_O_3_]^−^, 179.0352 [M−H−C_7_H_10_O_5_]^−^, 135.0449 [M−H−C_7_H_10_O_5_−CO_2_]^−^
5^c^ (peak 4)	20.341	C_16_H_24_O_10_	Loganic acid	375.1290 [M−H]^−^	− 1.8	213.0769 [M−H−Glu]^−^, 169.0879, 112.9856
6^a^ (peak 5)	20.505	C_7_H_6_O_3_	*p*-hydroxybenzoic acid	137.0244 [M−H]^−^	− 0.1	108.0219, 93.0348
7^a,d^ (peak 15)	23.840	C_16_H_18_O_9_	Cryptohlorogenic acid	353.0876 [M−H]^−^	− 0.6	191.0560 [M−H−C_9_H_6_O_3_]^−^, 179.0346 [M−H−C_7_H_10_O_5_]^−^, 173.0455 [M−H−C_9_H_6_O_3_−H_2_O]^−^, 135.0454 [M−H−C_7_H_10_O_5_−CO_2_]^−^
8^b^ (peak 7)	23.905	C_20_H_30_O_13_	Kelampayoside A	477.1610 [M−H]^−^	− 0.8	375.0696, 293.0880 [M−H−C_9_H_12_O_4_]^−^, 206.9728
9^a,d^ (peak 16)	25.304	C_16_H_18_O_9_	Chlorogenic acid	353.0873 [M−H]^−^	− 1.4	191.0561 [M−H−C_9_H_6_O_3_]^−^
10^c,d^ (peak 8)	26.211	C_16_H_22_O_9_	Sweroside	359.1337 [M+H]^+^	0.1	197.0805 [M+H−Glu]^+^, 179.0703 [M+H−Glu−H_2_O]^+^, 127.0390 [M+H−Glu−C_4_H_6_O]^+^
11	29.558	C_26_H_30_N_2_O_10_	Unknown	531.1976 [M+H]^+^	0.5	369.1449, 281.0551, 233.0673, 241.1749
12^b^	30.041	C_26_H_34_N_2_O_9_	Naucleamide A-10-*O*-*β*-d-glucopyranoside	519.2332 [M+H]^+^	− 0.6	357.1808 [M+H−Glu]^+^, 339.1703 [M+H−Glu−H_2_O]^+^
13^b^	31.573	C_26_H_30_N_2_O_9_	10-hydroxystrictosamide (10-hydroxy vincosamide)	515.2029 [M+H]^+^	1.0	353.1497 [M+H−Glu]^+^, 283.1071 [M+H−Glu−C_4_H_6_O]^+^
14^b^	32.350	C_26_H_28_N_2_O_9_	3-epi-pumiloside	513.1872 [M+H]^+^	0.9	351.1354 [M+H−Glu]^+^, 333.0861 [M+H−Glu−H_2_O]^+^, 281.0537 [M+H−Glu−C_4_H_6_O]^+^
15^b^	34.338	C_28_H_34_N_2_O_11_	5-*β*-carboxystrictoside	575.2239 [M+H]^+^	0.6	413.1709 [M+H−Glu]^+^, 395.1608 [M+H−Glu−H_2_O]^+^, 343.1288 [M+H−Glu−C_4_H_6_O]^+^
16^b^ (peak 9)	35.349	C_26_H_28_N_2_O_9_	Pumiloside	513.1874 [M+H]^+^	1.3	351.1334 [M+H−Glu]^+^, 333.1233 [M+H−Glu−H_2_O]^+^, 281.0922 [M+H−Glu−C_4_H_6_O]^+^
17 (peak 11)	38.786	C_26_H_30_N_2_O_10_	Unknown	531.1976 [M+H]^+^	0.5	369.1441, 299.1028,281.0507
18^b,d^ (peak 12)	44.881	C_26_H_30_N_2_O_8_	Strictosamide	499.2085 [M+H]^+^	2.0	337.1538 [M+H−Glu]^+^, 319.1447 [M+H−Glu−H_2_O]^+^, 267.1129 [M+H−Glu−C_4_H_6_O]^+^
19^b^	45.048	C_27_H_32_N_2_O_10_	3*α*, 5*α*-tetrahydrodeoxycordifoline lactam	543.1975 [M−H]^−^	− 1.7	497.1941 [M−H−CH_2_O_2_]^−^, 335.1398 [M−H−Glu−CH_2_O_2_]^−^ 317.1290 [M−H−Glu−CH_2_O_2_−H_2_O]^−^, 265.0979 [M−H−Glu−CH_2_O_2_−H_2_O−C_4_H_4_]^−^
20	46.146	C_24_H_23_N_3_O_4_	Unknown	418.1761 [M+H]^+^	− 0.1	400.2261, 332.1396, 218.1420
21^b^	47.449	C_20_H_17_N_3_O_2_	Angustoline	332.1383 [M+H]^+^	− 3.2	–
22	49.228	C_21_H_30_N_2_O_7_	Unknown	390.1779 [M + H]^+^	− 1.6	327.1983, 158.1519, 133.0844
23^b,d^ (peak 13)	49.480	C_26_H_30_N_2_O_8_	Vincosamide	499.2065 [M+H]^+^	− 2.0	337.1551 [M+H−Glu]^+^, 319.1427 [M+H−Glu−H_2_O]^+^, 267.1131 [M+H−Glu−C_4_H_6_O]^+^

Peaks indicated the chromatographic peaks shown on the HPLC chromatogram

^a^Represented phenolic acids

^b^Represented alkaloids

^c^Represented iridoids

^d^Indicated compounds identified by comparing with the reference substances

The MS data of reference substances was summarized in Additional file 1. Seven compounds in Danmu preparations which were identified by comparing the HPLC retention times and MS data with those of the reference substances were marked in Table 2. For example, sweroside (reference substances) was detected in positive ion mode at 26.182 min with the m/z of 359.1335 (C_16_H_22_O_9_), and the MS/MS data were the m/z of 197.0806 [M+H−Glu]^+^, 179.0707 [M+H−Glu−H_2_O]^+^ and 127.0391 [M+H−Glu−C_4_H_6_O]^+^. The compound 10 in Table 2 was detected in positive ion mode at 26.211 min with the m/z of 359.1337 (C_16_H_22_O_9_), and the MS/MS data were the m/z of 197.0805 [M+H−Glu]^+^, 179.0703 [M+H−Glu−H_2_O]^+^, 127.0390 [M+H−Glu−C_4_H_6_O]^+^. Therefore, the compounds 10 was identified as sweroside. Based on the above identification process, seven compounds were characterized as protocatechuic acid, neochlorogenic acid, cryptochlorogenic acid, chlorogenic acid, sweroside, strictosamide and vincosamide.

The remaining compounds were tentatively assigned based on the previous literatures [25–29]. For example, the compounds 12 gave the 519.2332 [M+H]^+^ ions in positive mode and further revealed 357.1808 [M+H−Glu]^+^, 339.1703 [M+H−Glu−H_2_O]^+^ fragment ions, which corresponded to the MS data of Naucleamide A-10-*O*-*β*-d-glucopyranoside reported in the literature [27]. In the ESI–MS spectra of the compound 21, the 332.1383 [M+H]^+^ (C_20_H_17_N_3_O_2)_ was observed, while no MS/MS fragment ion was obtained. Referring to the previous literatures [25, 26], the molecular weight of angustoline was consistent with that of the compound 21. By extracting the exact molecular weight on the mass spectrum, only compound 21 at 47.449 min was found. Thus, the compound 21 was inferred as angustoline.

### HPLC method validation

The proposed method was validated for the simultaneous determination of protocatechuic acid, neochlorogenic acid, cryptochlorogenic acid, chlorogenic acid, sweroside, strictosamide and vincosamide in Danmu preparations. All the calibration curves exhibited good linearity (*r* > 0.9995) over the test ranges, and the values of LODs and LOQs were in the ranges of 0.039–0.086 and 0.098–0.275 μg/mL, respectively. The RSD values of the intra- and inter-day variations for seven components were less than 1.91% and 1.77%, respectively. The RSD values of repeatability ranged from 0.50 to 2.98%. The RSD values of stability were in the range of 0.40% to 2.93%. The overall recoveries varied between 94.67 and 103.64%, with RSD values less than 3.62%. These results are shown in Tables 3, 4 and 5, which indicated that this method was accurate, simple and reliable for the determination of seven chemical markers in Danmu preparations.

**Table 3:** Linear regression equation, linear ranges, LODs, and LOQs

Compounds	Regression equation	Linear ranges (μg/mL)	*r*	LOD (μg/mL)	LOQ (μg/mL)
Protocatechuic acid	y = 24.468x − 0.078	0.62–36.90	1.0000	0.040	0.102
Neochlorogenic acid	y = 16.211x + 1.285	0.65–39.00	0.9999	0.059	0.149
Cryptochlorogenic acid	y = 15.700x − 2.045	0.70–42.00	1.0000	0.041	0.126
Chlorogenic acid	y = 16.345x − 2.202	0.54–32.40	1.0000	0.047	0.134
Sweroside	y = 14.224x + 0.310	0.59–35.10	1.0000	0.086	0.201
Strictosamide	y = 16.580x − 0.526	2.45–147.00	1.0000	0.077	0.275
Vincosamide	y = 17.197x − 0.580	0.64–38.10	0.9995	0.039	0.098

**Table 4:** The results of precision of seven compounds in Danmu preparations

Compounds	Intra-day (RSD%, n = 5)			Inter-day (RSD%, n = 6)		
	Low	Middle	High	Low	Middle	High
Protocatechuic acid	0.67	0.34	0.70	1.62	0.14	1.23
Neochlorogenic acid	1.04	1.28	0.78	1.54	1.77	0.99
Cryptochlorogenic acid	1.70	0.66	0.41	1.25	0.43	0.90
Chlorogenic acid	1.23	1.42	0.57	1.08	1.11	0.52
Sweroside	1.39	0.27	0.64	1.39	0.16	1.37
Strictosamide	1.17	0.18	0.58	1.48	0.16	1.00
Vincosamide	1.91	0.44	0.50	1.36	0.51	1.09

**Table 5:** Repeatability, stability, and recovery of seven compounds in Danmu preparations

Compounds	Danmu Capsule			Danmu Syrup		
	Repeatability (RSD %, n = 6)	Stability (RSD%, n = 6)	Recovery, mean (RSD%, n = 6)	Repeatability (RSD%, n = 6)	Stability (RSD%, n = 6)	Recovery, mean (RSD%, n = 6)
Protocatechuic acid	0.78	0.82	103.27 (1.23)	1.93	0.88	102.98 (1.43)
Neochlorogenic acid	2.76	2.38	94.67 (1.05)	2.46	2.53	98.77 (3.62)
Cryptochlorogenic acid	2.47	2.93	99.65 (2.01)	2.98	2.77	100.09 (2.34)
Chlorogenic acid	1.18	1.65	100.34 (1.54)	2.86	0.69	102.38 (1.17)
Sweroside	1.47	2.38	100.97 (1.27)	2.09	2.33	103.64 (1.15)
Strictosamide	0.50	0.73	97.81 (1.08)	1.69	0.44	95.34 (1.29)
Vincosamide	1.36	2.84	98.86 (1.64)	2.87	0.40	102.42 (1.02)

### Quantitative determination

**Fig. 4:**
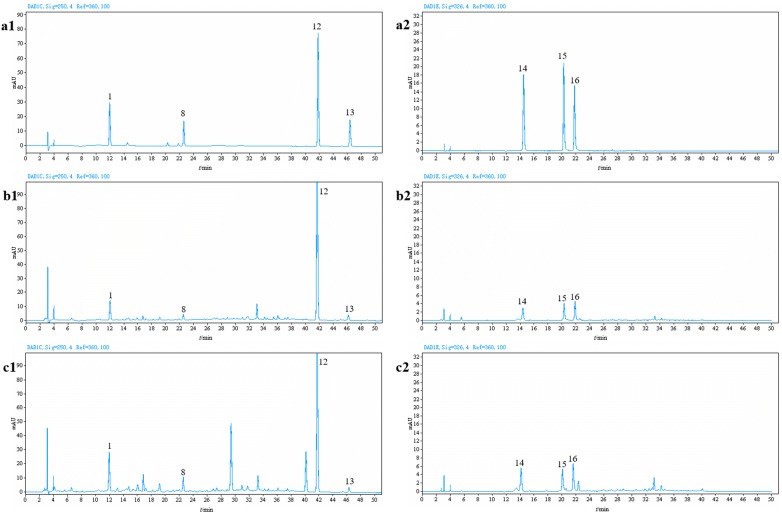
Representative HPLC chromatograms of mixed standards (**a1**, **a2**), Danmu Capsule (**b1**, **b2**) and Danmu Syrup (**c1**, **c2**) at wavelengths of 250 nm (**a1**, **b1**, **c1**) and 326 nm (**a2**, **b2**, **c2**). The peak numbers represented protocatechuic acid (1), neochlorogenic acid (14), cryptochlorogenic acid (15), chlorogenic acid (16) and sweroside (8), strictosamide (12) and vincosamide (13)

The developed HPLC–DAD method was applied to quantitative analysis of protocatechuic acid, neochlorogenic acid, cryptochlorogenic acid, chlorogenic acid, sweroside, strictosamide and vincosamide in the samples of Danmu preparations containing 12 batches of Danmu Capsule and 13 batches of Danmu Syrup (Fig. 4). Each sample was determined in duplicate, and the results are summarized in Table 1. As a result, it was found that strictosamide was the predominant constituent in Danmu preparations among these markers. The contents of strictosamide in different Danmu Capsule samples (43,681.20–99,652.49 μg/g) varied more greatly than that in different Danmu Syrup samples (1567.83–2427.25 μg/mL). In addition, the contents of protocatechuic acid, which was another major constituent in the capsule samples and syrup samples, were in the ranges of 2633.01–7739.78 μg/g and 192.05–448.71 μg/mL, respectively. As an iridoid, the contents of sweroside in Danmu Capsule and Danmu Syrup were 1573.82–2789.81 μg/g and 70.32–182.81 μg/mL, respectively. It is well known that different growth environments, harvesting seasons, processing, storage methods and storage times all affect the quality of TCM and their preparations. This may be the reason for the difference in the contents of different batches of preparations. However, to ensure the safety and efficacy of clinical drugs, the content of each compound in the preparation should be strictly controlled. The results indicated that the method in this paper was stable and reliable and could provide a scientific basis for the quality control of these preparations.

## Discussion

To obtain satisfactory extraction efficiency, the extraction method (ultrasonic or reflux extraction), solvents (95% methanol, 70% methanol, 50% methanol or water), and extraction time (20 min, 30 min or 40 min) were investigated for Danmu Capsule (Additional file 2). The results showed that the total extracted amounts of the seven active ingredients were the largest when the solvent was 70% methanol. In addition, there was no significant difference between ultrasonic and reflux extraction based on the chromatograms. Finally, to save time and operate simply on the basis of guaranteeing the highest extraction rate, 0.1 g of sample powder from Danmu Capsule was extracted by ultrasonication with 100 mL 70% methanol for 30 min. For the Danmu Syrup, 1 mL sample solution was directly diluted to 25 mL with 70% methanol.

Different HPLC conditions (mobile phase, column temperature and ultraviolet wavelength) were optimized to obtain better separation (Additional file 3). There were more peaks on the chromatogram when the mobile phase was acetonitrile which was more suitable than methanol. Different mobile phase modifiers (including 0.1% formic acid, 10 mM ammonium formate and 0.1% ammonium hydroxide) were examined and compared. In addition, the results suggested that 0.1% formic acid was better at ensuring more peaks, suppressing peak tailing and increasing the symmetry of the peak shape. A temperature of 35 °C exhibited better peak separation in a comparison of different column temperatures (30, 35, and 40 °C). Based on the ultraviolet spectra of the seven components recorded from 200 to 400 nm, the final wavelengths for monitoring were 250 nm and 326 nm. Among them, 250 nm was the best wavelength for HPLC fingerprints, where the baseline was stable and more chromatographic peaks can be obtained.

Comparing the standard fingerprints of Danmu Capsule and Danmu Syrup, it can be found that after excluding the interference of excipients, the two are very similar and both have 13 common peaks. In order to verify the HPLC fingerprint method, the strictosamide (peak 12) was selected as the reference peak, and the relative retention time and relative peak areas of all common peaks were calculated (Additional file 4). In the precision test, the same sample solution was analyzed by five consecutive injections. The RSD values of the 13 common peaks for the relative retention time and the relative peak area were < 1.58% and 4.50%, respectively. Besides, the same sample solution was analyzed at 0, 4, 8, 12 and 24 h and the RSD values of the relative retention time and the relative peak area were 0.01–1.14% and 0.25–4.74%, which indicated that the sample solution was stable within 24 h. For the repeatability test, the RSD values of the relative retention time and the relative peak area were 0.01–0.91% and 0.40–3.92%. The method verification results showed that the established qualitative analysis method of fingerprint was feasible and reliable. According to the similarity calculation results, the similarity values of Danmu Capsule and Danmu Syrup were both above 0.993 compared with the reference chromatogram, indicating a good consistency among batch-to-batch of the two preparations in qualitative evaluation. Therefore, the established fingerprint could be used as a practical qualitative identification tool for Danmu preparations, and could be used to evaluate the standardization, uniformity and stability of the preparation process. However, to further explore the quality differences among batches, the quantitative analysis needed be performed.

The research results showed that the content of each component in the Danmu Capsule and Danmu Syrup was quite different. It was speculated that this difference may be due to the following points: First, the preparations were made from the fresh stem of *Nauclea officinalis*, which can be harvested in all seasons. Wang et al. [30] analyzed the differences in the content of strictosamide in cultivated and wild Danmu from different places and at different plant ages, and the results showed that the quality of Danmu grown in different cities, counties and towns was quite different. The environment had a significant effect on the accumulation of strictosamide in Danmu. The mass fraction of strictosamide in Danmu planted for 2–5 years increased year by year, and the content of strictosamide over 4 years exceeded 1.6%, with the highest at 5 years. Second, the processing process also had a great impact on the content of the ingredients in the preparations. Two Danmu preparations were prepared by boiling in water three times, but their filtration times and densities were different. Higher clarity and homogeneity were required in the syrup, so its content distribution may have been more uniform. In addition, the syrup needed to undergo a sterilization process, and this process may have caused a certain level of content reduction. Finally, because fresh Danmu is made into preparations within half a month after harvest, the storage method and storage time had a great impact on the preparations, not only causing a large change in the moisture content but also causing the transfer or degradation of the ingredients in the medicinal materials. The above reasons may be the cause of the wide distribution range of the content of each component in the preparations. Therefore, it is important to standardize the collection, processing, production process and storage method based on quality control.

## Conclusions

In this study, the chromatographic fingerprints of Danmu preparations were established as a practical qualitative identification tool for the first time. A total of 23 compounds was identified by HPLC–Q-TOF-MS, seven of which were simultaneously determined, namely, protocatechuic acid, neochlorogenic acid, cryptochlorogenic acid, chlorogenic acid, sweroside, strictosamide and vincosamide. According to the results of the content determination, the growth environments, harvesting seasons, processing, storage methods and storage time have a great impact on the contents of the ingredients in the preparations. Therefore, it is significant to standardize the harvesting, processing, and production processes on the basis of quality inspection. In conclusion, the HPLC fingerprints established in this experiment supplies a scientific basis for producing Danmu preparations of stable quality, and the results of assay of Danmu Syrup and Danmu Capsule provides a data reference for establishing quality standards for large-scale production in enterprises.

## Supplementary information

**Additional file 1.** The MS data of reference substances.

**Additional file 2.** Optimization of the extraction method.

**Additional file 3.** Optimization of the HPLC conditions.

**Additional file 4.** Precision, stability and repeatability test for identification of common peaks by HPLC fingerprint.

## Data Availability

The research data generated from this study is included within the article.

## Abbreviations

HPLC: High-performance liquid chromatography
DAD: Diode array detection
HPLC–Q-TOF-MS: High-performance liquid chromatography coupled with quadrupole-time-of-flight high-definition mass spectrometry
TCM: Traditional Chinese medicine
ESI: Electrospray ionization
RSD: Relative standard deviations
LOD: Limit of detection
LOQ: Limit of quantitation
