# Supplementary material for: Chromatographic fingerprints analysis and determination of seven components in Danmu preparations by HPLC–DAD/QTOF-MS
Source: Chin Med. 2020 Feb 18;15:19. doi: 10.1186/s13020-020-00301-5 (PMC7027017; doi:10.1186/s13020-020-00301-5)
Supplement: Supplementary file 1 — Additional file 1. The MS data of reference substances. [file 13020_2020_301_MOESM1_ESM.pdf]

Table S1-1. The MS data of reference substances

t (min)	Molecular formula	Identification	$[M + H]^+$ / $[M – H]^-$ (m/z)		Fragmentations (m/z)
			Measured mass (m/z)	Error (ppm)	
17.140	$C_7H_6O_4$	Protocatechuic acid	153.0194 $[M – H]^-$	0.4	109.0294 $[M – CO_2 – H]^-$, 91.0191 $[M – H – CO_2 – H_2O]^-$, 81.0347
18.842	$C_{16}H_{18}O_9$	Neochlorogenic acid	353.0874$[M – H]^-$	– 1.1	191.0562 $[M – H – C_9H_6O_3]^-$, 179.0351 $[M – H – C_7H_{10}O_5]^-$, 135.0453 $[M – H – C_7H_{10}O_5 – CO_2]^-$
23.865	$C_{16}H_{18}O_9$	Cryptohlorogenic acid	353.0874 $[M – H]^-$	– 0.4	191.0560 $[M – H – C_9H_6O_3]^-$, 179.0349 $[M – H – C_7H_{10}O_5]^-$, 173.0453 $[M – H – C_9H_6O_3 – H_2O]^-$, 135.0454 $[M – H – C_7H_{10}O_5 – CO_2]^-$
25.325	$C_{16}H_{18}O_9$	Chlorogenic acid	353.0875 $[M – H]^-$	– 0.4	191.0562 $[M – H – C_9H_6O_3]^-$
26.182	$C_{16}H_{22}O_9$	Sweroside	359.1335 $[M + H]^+$	– 0.4	197.0806 $[M + H – Glu]^+$, 179.0707 $[M + H – Glu – H_2O]^+$, 127.0391 $[M + H – Glu – C_4H_6O]^+$
44.838	$C_{26}H_{30}N_2O_8$	Strictosamide	499.2072 $[M + H]^+$	– 0.6	337.1536 $[M + H – Glu]^+$, 319.1445 $[M + H – Glu – H_2O]^+$, 267.1125 $[M + H – Glu – C_4H_6O]^+$
49.480	$C_{26}H_{30}N_2O_8$	Vincosamide	499.2074 $[M + H]^+$	– 2.0	337.1544 $[M + H – Glu]^+$, 319.1435 $[M + H – Glu – H_2O]^+$, 267.1129 $[M + H – Glu – C_4H_6O]^+$
