# Supplementary material for: Chromatographic fingerprints analysis and determination of seven components in Danmu preparations by HPLC–DAD/QTOF-MS
Source: Chin Med. 2020 Feb 18;15:19. doi: 10.1186/s13020-020-00301-5 (PMC7027017; doi:10.1186/s13020-020-00301-5)
Supplement: Supplementary file 2 — Additional file 2. Optimization of the extraction method. [file 13020_2020_301_MOESM2_ESM.pdf]

Table S2-1. The contents (μg/g) of seven investigated compounds in Danmu Capsule by different extraction methods

Conditions	Protocatechuic acid	Neochlorogenic acid	Cryptochlorogenic acid	Chlorogenic acid	Sweroside	Strictosamide	Vincosamide	Total
Ultrasonic	5003.44	2467.16	2892.37	3242.86	1857.45	47470.98	2691.72	65625.98
Heating reflux	5082.27	2278.62	3055.13	3368.56	1819.96	47791.34	2432.74	65828.62

Table S2-2. The contents (μg/g) of seven investigated compounds in Danmu Capsule by different extraction solvents

Conditions	Protocatechuic acid	Neochlorogenic acid	Cryptochlorogenic acid	Chlorogenic acid	Sweroside	Strictosamide	Vincosamide	Total
95% methanol	4791.89	1604.38	1724.88	2078.74	1692.81	46818.91	2463.35	61174.96
70% methanol	5003.44	2467.16	2892.37	3242.86	1857.45	47470.98	2691.72	65625.98
50% methanol	4865.49	2498.75	2436.06	2466.29	1734.44	45599.58	2393.34	61993.95
Water	4983.71	2948.86	2671.30	3028.59	1735.39	43251.26	2331.54	60950.65

Table S2-3. The contents (μg/g) of seven investigated compounds in Danmu Capsule by different extraction times

Conditions	Protocatechuic acid	Neochlorogenic acid	Cryptochlorogenic acid	Chlorogenic acid	Sweroside	Strictosamide	Vincosamide	Total
20 min	4979.21	2352.07	2882.69	3359.38	1820.83	45926.05	2291.90	63612.13
30 min	5003.44	2467.16	2892.37	3242.86	1857.45	47470.98	2691.72	65625.98
40 min	5088.77	2293.04	2816.19	3299.49	1923.11	47251.45	2509.30	65181.35
