# Supplementary material for: Chromatographic fingerprints analysis and determination of seven components in Danmu preparations by HPLC–DAD/QTOF-MS
Source: Chin Med. 2020 Feb 18;15:19. doi: 10.1186/s13020-020-00301-5 (PMC7027017; doi:10.1186/s13020-020-00301-5)
Supplement: Supplementary file 3 — Additional file 3. Optimization of the HPLC conditions. [file 13020_2020_301_MOESM3_ESM.pdf]

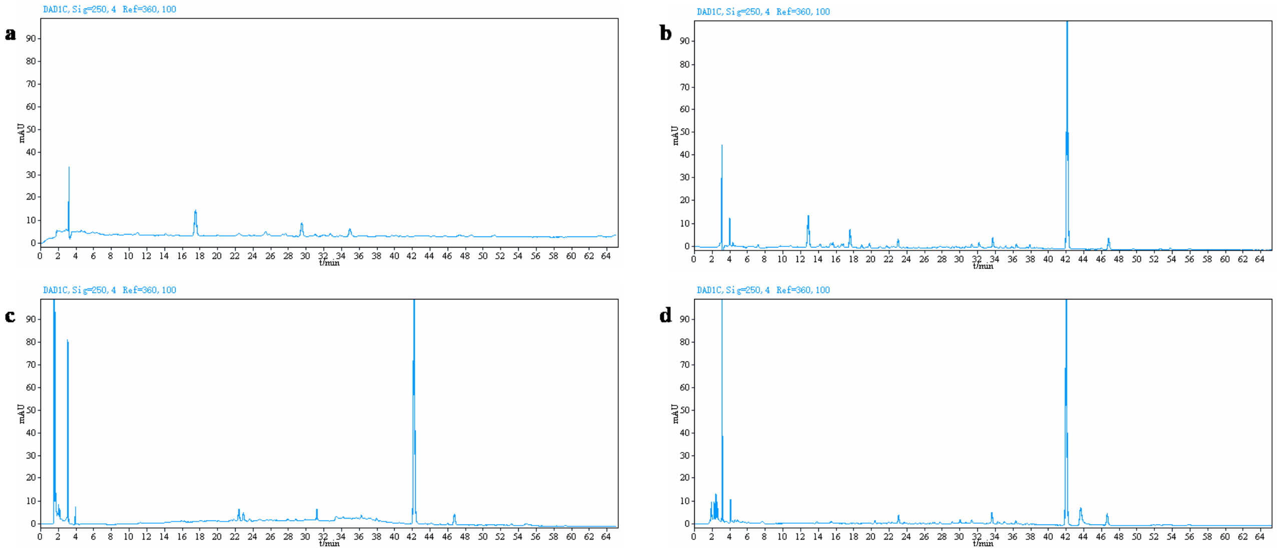

Fig. S3-1 Optimization of different mobile phase. **a** 0.1% formic acid aqueous solution - methanol; **b** 0.1% formic acid aqueous solution - acetonitrile; **c** 0.1% ammonium hydroxide aqueous solution - acetonitrile; **d** 10 mM ammonium formate aqueous solution – acetonitrile

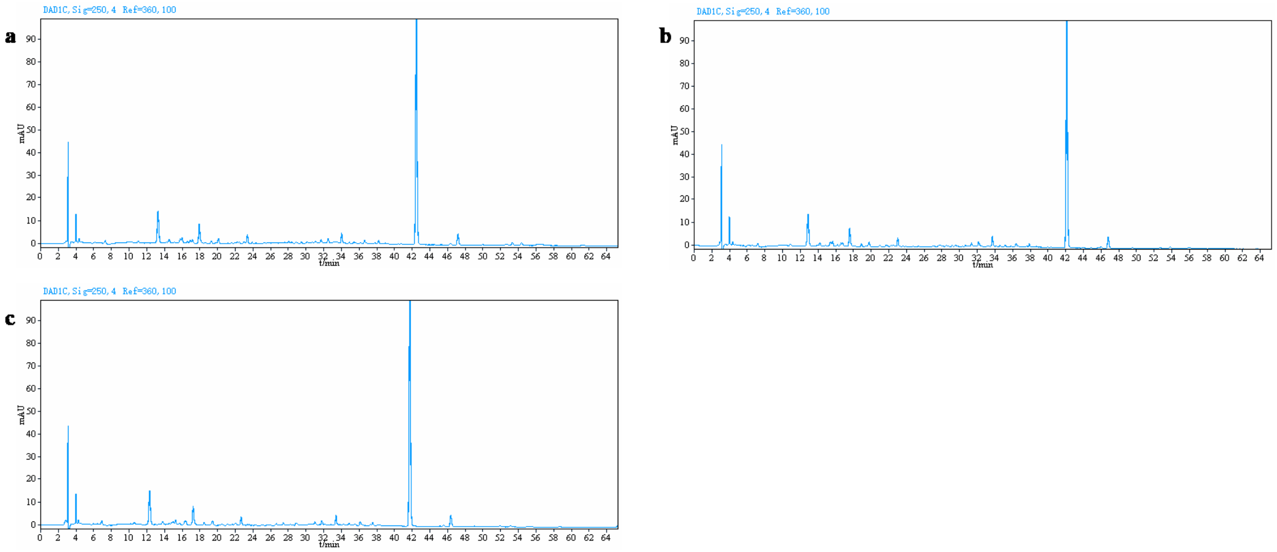

Fig. S3-2 Optimization of column temperature. **a** 30 °C; **b** 35 °C; **c** 40 °C.

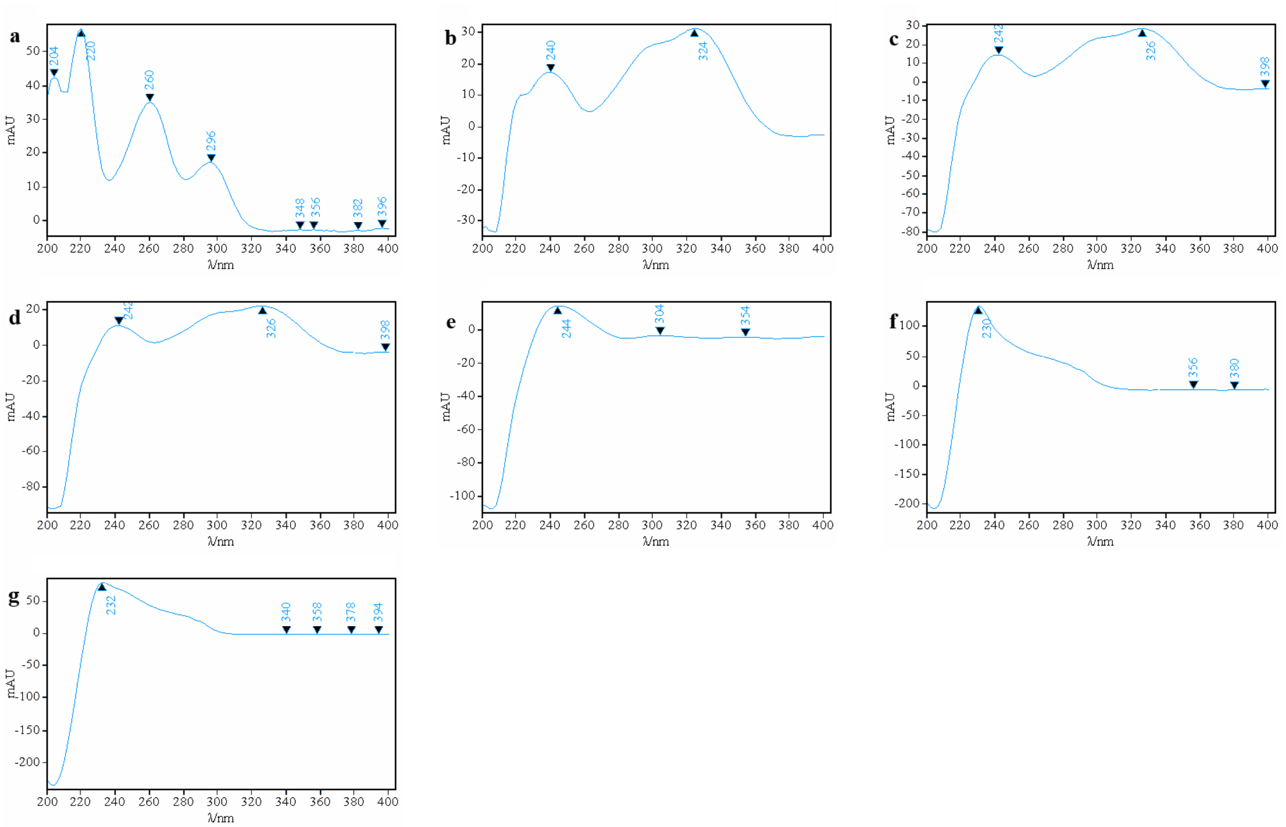

Fig. S3-3 The UV spectra of all seven components from 200 to 400 nm. **a** protocatechuic acid; **b** neochlorogenic acid; **c** cryptochlorogenic acid; **d** chlorogenic acid; **e** sweroside; **f** strictosamide; **g** vincosamide.
