# Supplementary material for: Chromatographic fingerprints analysis and determination of seven components in Danmu preparations by HPLC–DAD/QTOF-MS
Source: Chin Med. 2020 Feb 18;15:19. doi: 10.1186/s13020-020-00301-5 (PMC7027017; doi:10.1186/s13020-020-00301-5)
Supplement: Supplementary file 4 — Additional file 4. Precision, stability and repeatability test for identification of common peaks by HPLC fingerprint. [file 13020_2020_301_MOESM4_ESM.pdf]

Table S4-1. Precision test for identification of common peaks by HPLC fingerprint (Danmu Capsule)

Peak	Relative retention time					RSD	Relative peak area					RSD
no.	S1	S2	S3	S4	S5	(%)	S1	S2	S3	S4	S5	(%)
1	0.2945	0.2936	0.3005	0.2940	0.2970	0.98	0.1598	0.1610	0.1613	0.1610	0.1623	0.56
2	0.3261	0.3252	0.3335	0.3258	0.3293	1.05	0.0124	0.0124	0.0129	0.0121	0.0121	2.53
3	0.3896	0.3893	0.3934	0.3895	0.3914	0.45	0.0236	0.0232	0.0237	0.0233	0.0246	2.34
4	0.4090	0.4089	0.4127	0.4092	0.4107	0.40	0.0668	0.0688	0.0709	0.0663	0.0690	2.69
5	0.4186	0.4183	0.4226	0.4186	0.4205	0.43	0.0126	0.0135	0.0141	0.0129	0.0137	4.49
6	0.4419	0.4416	0.4449	0.4418	0.4432	0.32	0.0108	0.0108	0.0106	0.0109	0.0109	1.10
7	0.4640	0.4637	0.4664	0.4640	0.4649	0.24	0.0199	0.0191	0.0191	0.0192	0.0188	2.04
8	0.5443	0.5440	0.5461	0.5443	0.5448	0.16	0.0337	0.0328	0.0326	0.0356	0.0316	4.50
9	0.7941	0.7944	0.7944	0.7942	0.7939	0.03	0.0369	0.0360	0.0372	0.0375	0.0374	1.59
10	0.8656	0.8658	0.8658	0.8658	0.8653	0.03	0.0127	0.0128	0.0127	0.0122	0.0123	2.13
11	0.8987	0.8987	0.8988	0.8989	0.8983	0.03	0.0115	0.0114	0.0119	0.0117	0.0118	1.74
12	1.0000	1.0000	1.0000	1.0000	1.0000	-	1.0000	1.0000	1.0000	1.0000	1.0000	-
13	1.1084	1.1081	1.1082	1.1082	1.1087	0.02	0.0566	0.0563	0.0568	0.0563	0.0567	0.44

Table S4-2. Precision test for identification of common peaks by HPLC fingerprint (Danmu Syrup)

Peak	Relative retention time					RSD	Relative peak area					RSD
no.	S1	S2	S3	S4	S5	(%)	S1	S2	S3	S4	S5	(%)
1	0.2993	0.2883	0.2943	0.2901	0.2891	1.58	0.1986	0.1982	0.1980	0.1987	0.1980	0.17
2	0.3220	0.3183	0.3169	0.3208	0.3195	0.63	0.0190	0.0188	0.0189	0.0186	0.0186	0.96
3	0.3878	0.3907	0.3900	0.3874	0.3869	0.43	0.0373	0.0371	0.0408	0.0368	0.0383	4.29
4	0.4075	0.4100	0.4095	0.4071	0.4065	0.38	0.0731	0.0734	0.0744	0.0726	0.0708	1.85
5	0.4166	0.4195	0.4190	0.4161	0.4155	0.43	0.0212	0.0209	0.0211	0.0210	0.0203	1.62
6	0.4402	0.4425	0.4421	0.4396	0.4395	0.32	0.0079	0.0080	0.0078	0.0078	0.0078	0.81
7	0.4627	0.4645	0.4643	0.4627	0.4628	0.19	0.0410	0.0411	0.0399	0.0405	0.0405	1.23
8	0.5430	0.5444	0.5444	0.5431	0.5432	0.13	0.0689	0.0689	0.0687	0.0680	0.0693	0.66
9	0.7942	0.7950	0.7945	0.7948	0.7946	0.04	0.0578	0.0598	0.0582	0.0579	0.0597	1.66
10	0.8655	0.8659	0.8657	0.8659	0.8658	0.02	0.0122	0.0124	0.0124	0.0122	0.0123	0.82
11	0.8986	0.8988	0.8987	0.8997	0.8996	0.06	0.0099	0.0105	0.0103	0.0103	0.0103	2.06
12	1.0000	1.0000	1.0000	1.0000	1.0000	-	1.0000	1.0000	1.0000	1.0000	1.0000	-
13	1.1085	1.1082	1.1083	1.1081	1.1082	0.01	0.0295	0.0301	0.0292	0.0296	0.0301	1.26

Table S4-3. Stability test for identification of common peaks by HPLC fingerprint (Danmu Capsule)

Peak	Relative retention time					RSD	Relative peak area					RSD
no.	S1	S2	S3	S4	S5	(%)	S1	S2	S3	S4	S5	(%)
1	0.2844	0.2857	0.2790	0.2844	0.2839	0.92	0.1578	0.1570	0.1586	0.1545	0.1556	1.04
2	0.3095	0.3093	0.3020	0.3097	0.3106	1.14	0.0137	0.0129	0.0124	0.0129	0.0129	3.85
3	0.3830	0.3825	0.3775	0.3826	0.3823	0.61	0.0247	0.0226	0.0221	0.0221	0.0230	4.61
4	0.4019	0.4017	0.4048	0.4016	0.4015	0.35	0.0700	0.0675	0.0675	0.0666	0.0678	1.88
5	0.4097	0.4096	0.4048	0.4094	0.4096	0.52	0.0113	0.0109	0.0114	0.0114	0.0124	4.74
6	0.4353	0.4347	0.4301	0.4346	0.4341	0.49	0.0112	0.0105	0.0106	0.0104	0.0106	2.74
7	0.4589	0.4583	0.4549	0.4586	0.4583	0.36	0.0207	0.0220	0.0202	0.0201	0.0213	3.84
8	0.5393	0.5403	0.5371	0.5402	0.5394	0.24	0.0338	0.0358	0.0328	0.0321	0.0343	4.23
9	0.7970	0.7965	0.7971	0.7976	0.7964	0.06	0.0400	0.0398	0.0382	0.0413	0.0390	2.91
10	0.8655	0.8653	0.8656	0.8658	0.8653	0.02	0.0153	0.0150	0.0151	0.0151	0.0149	0.87
11	0.8988	0.8988	0.8985	0.8987	0.8979	0.04	0.0122	0.0122	0.0121	0.0122	0.0122	0.45
12	1.0000	1.0000	1.0000	1.0000	1.0000	-	1.0000	1.0000	1.0000	1.0000	1.0000	-
13	1.1085	1.1082	1.1083	1.1081	1.1082	0.01	0.0295	0.0301	0.0292	0.0296	0.0301	1.26

Table S4-4. Stability test for identification of common peaks by HPLC fingerprint (Danmu Syrup)

Peak	Relative retention time					RSD	Relative peak area					RSD
no.	S1	S2	S3	S4	S5	(%)	S1	S2	S3	S4	S5	(%)
1	0.2841	0.2817	0.2805	0.2835	0.2820	0.51	0.1963	0.1978	0.2003	0.1997	0.2002	0.87
2	0.3092	0.3053	0.3038	0.3086	0.3066	0.74	0.0204	0.0195	0.0196	0.0202	0.0195	2.25
3	0.3813	0.3794	0.3784	0.3811	0.3802	0.32	0.0453	0.0446	0.0426	0.0452	0.0432	2.72
4	0.4010	0.3992	0.3986	0.4009	0.4003	0.26	0.0772	0.0757	0.0755	0.0768	0.0746	1.38
5	0.4106	0.4083	0.4076	0.4101	0.4093	0.30	0.0209	0.0217	0.0215	0.0213	0.0208	1.80
6	0.4341	0.4317	0.4313	0.4334	0.4329	0.26	0.0082	0.0080	0.0080	0.0082	0.0081	1.20
7	0.4578	0.4551	0.4548	0.4563	0.4563	0.26	0.0460	0.0446	0.0415	0.0456	0.0442	3.93
8	0.5393	0.5394	0.5385	0.5394	0.5401	0.10	0.0695	0.0655	0.0656	0.0672	0.0660	2.50
9	0.7944	0.7954	0.7952	0.7941	0.7954	0.08	0.0598	0.0597	0.0629	0.0598	0.0601	2.27
10	0.8656	0.8660	0.8655	0.8650	0.8659	0.04	0.0131	0.0133	0.0131	0.0129	0.0129	1.43
11	0.8982	0.8985	0.8980	0.8977	0.8982	0.03	0.0106	0.0111	0.0109	0.0111	0.0111	1.87
12	1.0000	1.0000	1.0000	1.0000	1.0000	-	1.0000	1.0000	1.0000	1.0000	1.0000	-
13	1.1090	1.1083	1.1087	1.1085	1.1082	0.03	0.0237	0.0236	0.0237	0.0238	0.0237	0.25

Table S4-5. Repeatability test for identification of common peaks by HPLC fingerprint (Danmu Capsule)

Peak no.	Relative retention time					RSD (%)	Relative peak area					RSD (%)
	S1	S2	S3	S4	S5		S1	S2	S3	S4	S5	
1	0.2854	0.2877	0.2844	0.2869	0.2886	0.59	0.1542	0.1549	0.1536	0.1548	0.1565	0.69
2	0.3128	0.3169	0.3126	0.3160	0.3194	0.91	0.0124	0.0119	0.0123	0.0118	0.0121	1.91
3	0.3826	0.3855	0.3834	0.3846	0.3865	0.41	0.0241	0.0241	0.0243	0.0236	0.0229	2.31
4	0.4018	0.4049	0.4025	0.4036	0.4057	0.40	0.0663	0.0657	0.0653	0.0655	0.0656	0.55
5	0.4099	0.4129	0.4102	0.4116	0.4138	0.41	0.0120	0.0123	0.0126	0.0130	0.0126	2.85
6	0.4343	0.4373	0.4351	0.4359	0.4379	0.34	0.0105	0.0106	0.0106	0.0105	0.0105	0.53
7	0.4579	0.4608	0.4591	0.4593	0.4607	0.27	0.0227	0.0215	0.0219	0.0219	0.0204	3.92
8	0.5392	0.5418	0.5405	0.5401	0.5416	0.20	0.0325	0.0319	0.0326	0.0326	0.0331	1.34
9	0.7967	0.7966	0.7967	0.7967	0.7966	0.01	0.0384	0.0364	0.0380	0.0378	0.0384	2.21
10	0.8654	0.8653	0.8655	0.8653	0.8656	0.02	0.0152	0.0154	0.0151	0.0151	0.0151	0.98
11	0.8982	0.8981	0.8981	0.8981	0.8983	0.01	0.0126	0.0122	0.0123	0.0121	0.0124	1.59
12	1.0000	1.0000	1.0000	1.0000	1.0000	-	1.0000	1.0000	1.0000	1.0000	1.0000	-
13	1.1091	1.1080	1.1088	1.1089	1.1090	0.04	0.0550	0.0530	0.0540	0.0532	0.0543	1.49

Table S4-6. Repeatability test for identification of common peaks by HPLC fingerprint (Danmu Syrup)

Peak no.	Relative retention time					RSD (%)	Relative peak area					RSD (%)
	S1	S2	S3	S4	S5		S1	S2	S3	S4	S5	
1	0.2854	0.2869	0.2867	0.2867	0.2831	0.56	0.1970	0.1979	0.1960	0.1973	0.1987	0.53
2	0.3130	0.3151	0.3147	0.3146	0.3097	0.71	0.0186	0.0187	0.0183	0.0187	0.0185	0.94
3	0.3839	0.3853	0.3847	0.3853	0.3821	0.34	0.0398	0.0398	0.0396	0.0403	0.0383	1.85
4	0.4028	0.4041	0.4037	0.4042	0.4013	0.30	0.0712	0.0715	0.0710	0.0726	0.0717	0.85
5	0.4109	0.4121	0.4120	0.4123	0.4093	0.31	0.0196	0.0201	0.0197	0.0200	0.0197	1.03
6	0.4351	0.4364	0.4361	0.4366	0.4342	0.24	0.0076	0.0077	0.0075	0.0078	0.0076	1.23
7	0.4586	0.4600	0.4595	0.4601	0.4581	0.19	0.0427	0.0429	0.0426	0.0432	0.0424	0.67
8	0.5400	0.5416	0.5406	0.5411	0.5399	0.13	0.0652	0.0649	0.0672	0.0649	0.0674	1.94
9	0.7962	0.7971	0.7970	0.7971	0.7971	0.05	0.0614	0.0648	0.0613	0.0649	0.0650	3.10
10	0.8649	0.8658	0.8655	0.8654	0.8655	0.04	0.0127	0.0126	0.0124	0.0127	0.0126	0.91
11	0.8977	0.8985	0.8983	0.8982	0.8984	0.04	0.0109	0.0109	0.0109	0.0108	0.0109	0.40
12	1.0000	1.0000	1.0000	1.0000	1.0000	-	1.0000	1.0000	1.0000	1.0000	1.0000	-
13	1.1099	1.1088	1.1088	1.1084	1.1090	0.05	0.0282	0.0267	0.0268	0.0269	0.0284	2.97
